# Metacognition across sensory modalities: Vision, warmth, and nociceptive pain

**DOI:** 10.1016/j.cognition.2019.01.018

**Published:** 2019-05

**Authors:** Brianna Beck, Valentina Peña-Vivas, Stephen Fleming, Patrick Haggard

**Affiliations:** a Institute of Cognitive Neuroscience, University College London, London, United Kingdom; b Wellcome Centre for Human Neuroimaging, University College London, London, United Kingdom; c Max Planck UCL Centre for Computational Psychiatry and Ageing Research, University College London, London, United Kingdom

**Keywords:** Affect, Arousal, Confidence, Nociception, Thermal, Visual

## Abstract

The distinctive experience of pain, beyond mere processing of nociceptive inputs, is much debated in psychology and neuroscience. One aspect of perceptual experience is captured by metacognition—the ability to monitor and evaluate one’s own mental processes. We investigated confidence in judgements about nociceptive pain (i.e. pain that arises from the activation of nociceptors by a noxious stimulus) to determine whether metacognitive processes contribute to the distinctiveness of the pain experience. Our participants made intensity judgements about noxious heat, innocuous warmth, and visual contrast (first-order, perceptual decisions) and rated their confidence in those judgements (second-order, metacognitive decisions). First-order task performance between modalities was balanced using adaptive staircase procedures. For each modality, we quantified metacognitive efficiency (meta-d’/d’)—the degree to which participants’ confidence reports were informed by the same evidence that contributed to their perceptual judgements—and metacognitive bias (mean confidence)—the participant’s tendency to report higher or lower confidence overall. We found no overall differences in metacognitive efficiency or mean confidence between modalities. Mean confidence ratings were highly correlated between all three tasks, reflecting stable inter-individual variability in metacognitive bias. However, metacognitive efficiency for pain varied independently of metacognitive efficiency for warmth and visual perception. That is, those participants who had higher metacognitive efficiency in the visual task also tended to have higher metacognitive efficiency in the warmth task, but not necessarily in the pain task. We thus suggest that some distinctive and idiosyncratic aspects of the pain experience may stem from additional variability at a metacognitive level. We further speculate that this additional variability may arise from the affective or arousal aspects of pain.

## 1 Introduction

Subjectivity is considered a fundamental aspect of the pain experience (e.g. Beecher, 1957, Beecher, 1965, Coghill et al., 2003, Guerit, 2012, Hyyppä, 1987, Koyama et al., 2005, Raij et al., 2005). One facet of subjective experience is metacognition—the ability to monitor and evaluate one’s own mental processes (Metcalfe & Shimamura, 1994). Metacognition can be measured by how closely confidence reports track the fidelity of the mental process in question. In perceptual decision-making tasks, people with high *metacognitive sensitivity* are more confident when they have made a correct judgement (i.e. when their perceptual decision accurately reflects the physical properties of a sensory stimulus) than when they have made an incorrect judgement. Independently of metacognitive sensitivity, a person might show a *metacognitive bias*, that is, a tendency to be over- or under-confident regardless of whether the judgement was correct. These measures jointly characterise how people evaluate their perceptual decisions. Applied to judgements about nociceptive pain—i.e. pain that arises from the activation of nociceptors by a noxious stimulus (IASP Task Force on Taxonomy, 2011)—metacognitive measures may shed light on some distinctive features of pain perception, such as its vividness and its variability, even when the physical properties of the evoking stimulus are held constant (Coghill et al., 2003, Nickel et al., 2017, Schulz et al., 2015, Woo et al., 2017).

There are several reasons to suspect that metacognition for nociceptive pain may differ from metacognition for other sensory modalities. First, nociception, like interoceptive senses, serves a primary role in body regulation and defence (Craig, 2002, Craig, 2003), rather than fine discrimination of stimulus attributes. Indeed, the first response to nociceptor activation is usually a reflexive defensive reaction (Ellrich et al., 1997, Skljarevski and Ramadan, 2002, Willer, 1977). Metacognitive oversight would benefit a sensory system tuned for discriminative precision because it allows for error correction and strategic behavioural adjustments in response to uncertainty (Redford, 2010, Yeung and Summerfield, 2012). In contrast, sensory systems that maintain homeostasis and facilitate quick defensive reactions must be able to function effectively without conscious cognitive control. Thus, metacognition may have less access to pain and to interoceptive senses than to sensory systems with fine discriminative capacities such as vision. Indeed, studies of interoceptive heartbeat perception have generally found poor metacognitive sensitivity to such signals (Azevedo et al., 2016, Garfinkel et al., 2015, Khalsa et al., 2008) and dissociations in metacognitive sensitivity between interoceptive and exteroceptive sensory modalities (Garfinkel et al., 2016).^2^ Nociceptive metacognition might be similarly dissociated from exteroceptive metacognition because a basic function of nociception is to defend the integrity of the body by allowing quick motor reactions.

Second, nociceptive pain elicits physiological arousal and affective responses in addition to sensory processes (Hilgard and Morgan, 1975, Lenox, 1970, Melzack and Casey, 1968, Rainville et al., 1999, Storm, 2008). Studies that induced changes in arousal through subliminal affective priming (Allen et al., 2016) and pharmacological manipulation (Hauser et al., 2017) suggested that arousal responses may reduce the tendency to adjust metacognitive judgements according to internal or external noise, although they disagreed on which aspect of metacognition (sensitivity or bias) was most affected. Additionally, some studies have reported that negatively-valenced material increased measures of confidence in perception (Koizumi, Mobbs, & Lau, 2016) and in subsequent recall (Schwartz, 2010, Zimmerman and Kelley, 2010), while others found no effect of negative valence on metacognition (D'Angelo and Humphreys, 2012, Jersakova et al., 2015). Though these studies offer mixed evidence on the relations between arousal, affect, and metacognition, they suggest that the negatively valenced and arousing qualities of nociceptive pain could alter the calibration of metacognitive judgements, perhaps yielding over-confidence in perceptual decisions.

We investigated how metacognitive access to nociception compares to thermoception, a sensory modality that also serves a regulatory role for the body, and to vision, a sensory modality with fine discriminative capacities that is widely studied in metacognition research. Participants made intensity discrimination judgements about three different kinds of stimuli: noxious heat (pain), innocuous warmth, and visual gratings (contrast). They also rated their confidence in those judgements. We quantified metacognitive access using the ratio meta-d’/d’. This represents the efficiency with which confidence ratings discriminate between ‘correct’ and ‘incorrect’ trials, while controlling for differences in perceptual sensitivity (Fleming, 2017, Maniscalco and Lau, 2012). To examine metacognitive bias, we also compared mean confidence ratings across these three modalities. We controlled task difficulty across participants and sensory modalities using an adaptive staircase procedure. Because both nociception and thermoception serve chiefly defensive and regulatory functions (Craig, 2002, Craig, 2003), we expected to find lower metacognitive efficiency scores for nociceptive pain and innocuous warmth discrimination tasks than for a visual contrast discrimination task. Further, we expected that individual differences in metacognitive efficiency would correlate across pain and warmth discrimination tasks, but that neither would correlate with metacognitive efficiency for visual contrast discrimination. Finally, we predicted higher confidence in judgements about pain, relative to judgements about warmth and visual contrast, because of the characteristic vividness and aversiveness of pain experiences.

## 2 Materials and methods

### 2.1 Participants

To determine sample size, we used sequential hypothesis testing with Bayes factors (Schönbrodt, Wagenmakers, Zehetleitner, & Perugini, 2017). We selected a minimum sample size of 24, and defined our stopping rule as the point at which the Bayes factors (BF_10_) for analyses of variance (ANOVAs) across our three conditions were higher than 3.00 (implying moderate support for the alternative hypothesis) or lower than 0.33 (implying moderate support for the null hypothesis; Jeffreys, 1961, Lee and Wagenmakers, 2013). We calculated Bayes factors after running 24 participants, and again after each additional 4 participants. Our stopping rule was reached at 36 participants (18 female, mean age = 24.50, range = 19–38). Sequential hypothesis testing with Bayes factors does not require corrections for multiple tests because the critical inference is based not on the probability of making a Type I error, but on a ratio (BF_10_) indicating how much more (or less) likely the data would be under the alternative hypothesis compared to the null hypothesis (Schönbrodt et al., 2017).

All participants had normal or corrected-to-normal vision, normal cutaneous sensation, and no history of neurological or psychiatric disorders by self-report. They gave written informed consent prior to the experiment, and were compensated for their time with a per-hour payment of £7.50 or 1 course credit. One participant chose not to complete the experiment, and another participant’s data were lost due to equipment failure. These incomplete datasets were not analysed. A third participant finished the experiment but performed at chance level on the innocuous warmth discrimination task, so that participant’s entire dataset was also excluded from all analyses. These participants were replaced with others in the final sample. The study was approved by the University College London Research Ethics Committee, and carried out in accordance with the provisions of the World Medical Association Declaration of Helsinki.

### 2.2 Materials

Visual stimuli and response prompts were generated in the Cogent 2000 toolbox (http://www.vislab.ucl.ac.uk/cogent.php) for MATLAB 8.5.0 (Mathworks Inc., Natick, MA, USA). The visual stimuli consisted of a central white fixation cross 2° across (luminance: 13.64 cd/m^2^) and Gabor gratings at 3° of visual angle (2.2 cycles per degree, 0.2° Gaussian envelope), presented at ±7.5° eccentricity from the fixation cross. The background was a uniform grey screen (luminance: 3.66 cd/m^2^). The stimuli were displayed on a 17″ LCD monitor (Dell E173FPb, Round Rock, TX, USA; 1280 × 1024 screen resolution, 75-Hz refresh rate). The display was gamma-calibrated using a CS-100A photometer (Konica Minolta, Tokyo, Japan).

Noxious and innocuous thermal stimuli were delivered using a computer-controlled Peltier thermode with a 13-mm diameter pen-shaped probe (Physitemp NTE-2A, Clifton, NJ, USA). The probe was affixed to a computer-controlled haptic device (PHANToM Premium 1.5, Geomagic, Morrisville, NC, USA) that was used to jitter stimulus position and to bring the probe into contact with the hand dorsum with a light force of 0.2 N. Skin temperature on the hand dorsum was monitored with a spot infrared thermometer (Precision Gold N85FR; Maplin Electronics, Rotherham, UK).

### 2.3 Procedure

All participants completed a perceptual intensity discrimination task in three different modalities: visual contrast, innocuous warmth, and nociceptive pain. Participants also completed a manipulation check in which they rated the painfulness of stimuli used in the nociceptive pain and innocuous warmth tasks, to confirm that the temperature ranges were perceived differently. These four tasks were completed in two experimental sessions on separate days. The second session was done within three days of the first session, and at the same time of day. Each session lasted about 1.5 h. The nociceptive pain and innocuous warmth discrimination tasks were always done in different sessions to minimise effects of habituation, sensitisation, or receptor fatigue from repeated thermal stimulation. The order of these tasks was counterbalanced across participants. The manipulation check was always done in the second session, after both the nociceptive pain and innocuous warmth discrimination tasks had been completed. The visual contrast discrimination task was done in the first session with either the nociceptive pain or the innocuous warmth discrimination task. Task order in the first session was counterbalanced across participants.

Each task consisted of 180 trials of a two-interval alternative forced choice (2IFC) judgement. Participants were given a short break after every 20 trials. The first 20 trials were considered a practice block, and were not included in any statistical analyses. Each trial consisted of a *reference stimulus*, which was presented at the same stimulus intensity (i.e. the same contrast or temperature) on every trial, and a *test stimulus*, whose intensity was adapted throughout the task using a continuous 2-down/1-up staircase procedure, in order to keep discrimination accuracy at approximately 70.7% (Levitt, 1971). The order and locations of the reference and target stimuli were counterbalanced across trials.

#### 2.3.1 Visual contrast discrimination

**Fig. 1:**
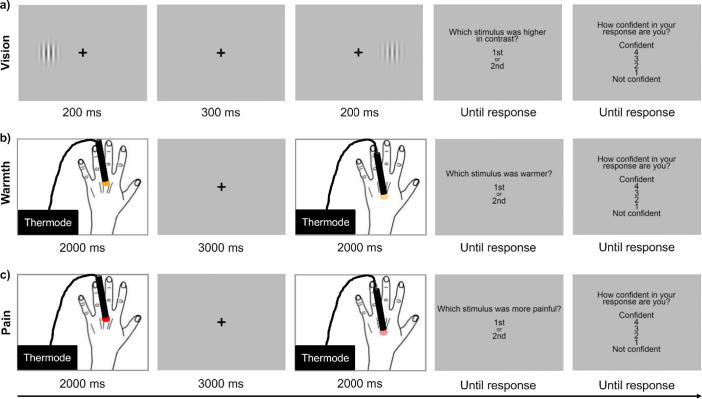
Examples of trials in (a) the visual contrast discrimination task, (b) the innocuous warmth discrimination task, and (c) the nociceptive pain discrimination task. For all three tasks, two stimuli of different intensities were presented sequentially in each trial. Participants made a forced choice intensity discrimination judgement, and then rated their confidence in that judgement on a 4-point scale.

Participants sat with their head in a chin rest approximately 57 cm from the screen. Each trial began with a central fixation cross (1000 ms), followed by two Gabor patches presented sequentially (200 ms each) with a 300-ms inter-stimulus interval (ISI). The first Gabor patch was presented either 7.5° to the left or 7.5° to the right of the fixation cross (pseudorandomly with equal probability across trials), and the second Gabor patch was presented in the other location, in order to mirror the spatial jittering procedure used for the innocuous warmth and noxious heat tasks (see 2.3.2, 2.3.3). After the offset of the second stimulus, a prompt appeared on the screen asking participants to report which stimulus was higher in contrast. Following their response, another prompt appeared asking them to report how confident they were in their response on a scale of 1 (*not confident*) to 4 (*confident*). Participants were encouraged to use the entire confidence scale over the course of the task. They used a numerical keypad to respond to both prompts (Fig. 1a).

The reference stimulus was always presented with 50% contrast. The test stimulus started at 70% and was adapted throughout the task based on performance. It was increased by 3% following an incorrect response and decreased by 3% following two consecutive correct responses.

#### 2.3.2 Innocuous warmth discrimination

Participants sat with their left hand placed palm down on the table in front of them. Prior to the task, the baseline skin temperature on their left hand dorsum was recorded (M = 31.04 °C, SD = 2.19 °C). Each trial began with a central fixation cross which remained on the screen until response prompts were displayed. The haptic device sequentially delivered two contact thermal stimuli (2000 ms each) to distinct locations on the left hand dorsum with a 3000-ms ISI. Stimulus location was jittered between four different locations on the hand dorsum to avoid peripheral effects such as receptor fatigue or persistent changes in skin temperature. The distance between these locations was adjusted for each participant based on hand size and shape, but was always at least 15 mm. After the offset of the second stimulus, a prompt appeared on the screen asking participants to report which stimulus was warmer. Then participants rated their confidence in their perceptual decision, as described in Section 2.3.1 above. Skin temperature on the left hand dorsum was monitored between blocks to ensure it had returned to the baseline skin temperature before starting the next block (mean change = 0.10 °C, SD = 0.27 °C).

The reference stimulus was always 38.0 °C. The target stimulus started at 40.0 °C and was adapted throughout the task based on performance. It was increased by 0.5 °C following an incorrect response and decreased by 0.5 °C following two consecutive correct responses. The test stimulus was never increased higher than 43.0 °C—even if a participant made an incorrect response when comparing a 43.0 °C test stimulus with the 38.0 °C reference stimulus—to avoid delivering stimuli in the noxious heat range.

#### 2.3.3 Nociceptive pain discrimination

The procedure of the nociceptive pain discrimination task was the same as the procedure for innocuous warmth discrimination (see Section 2.3.2), except that we used a higher temperature range of noxious heat for thermal stimulation, and participants reported which stimulus was more painful. The reference stimulus was always 45.0 °C (i.e. the normative heat pain threshold; Dyck et al., 1993, Yarnitsky et al., 1995). The target stimulus started at 47.0 °C and was adapted throughout the task based on performance. It was increased by 0.5 °C following an ‘incorrect’ response (i.e. an unexpected response based on noxious stimulus intensity) and decreased by 0.5 °C following two consecutive ‘correct’ responses (i.e. the expected response based on noxious stimulus intensity). The test stimulus was never increased higher than 50.0 °C as a precaution against skin damage. The baseline skin temperature on the left hand dorsum was recorded prior to the task (M = 31.24 °C, SD = 2.83 °C), and monitored between blocks to ensure it had returned to baseline before starting the next block (mean change = 0.17 °C, SD = 0.37 °C).

#### 2.3.4 Manipulation check for thermal stimuli

In each trial, a single thermal stimulus (2000 ms) was delivered to the left hand dorsum. The temperature of the stimulus was set to either the lowest temperature delivered in the nociceptive pain discrimination task (i.e. 45.0 °C) or the highest temperature delivered on any trial to each individual participant in the innocuous warmth discrimination task (M = 42.68 °C, SD = 0.54 °C). These temperatures were chosen to ensure that even the most similar stimuli delivered in the nociceptive pain and innocuous warmth discrimination tasks were perceived differently. After stimulus offset, a prompt appeared on the screen asking participants to report how painful the stimulus was on a scale of 1 (*not painful*) to 4 (*painful*). The brief task consisted of 20 trials—10 of each stimulus temperature—in a randomised order.

#### 2.3.5 Statistical analysis

First, we compared the percentage of correct responses between tasks using a Bayesian repeated measures ANOVA and Bayesian paired samples *t*-tests with default Cauchy priors (*t*-tests: *r* = 0.707; ANOVA: *r*_fixed_ = 1, *r*_random_ = 0.5) to check whether our staircase procedures were successful. Then we used participants’ 2IFC intensity judgements and confidence ratings to calculate signal detection theoretic measures of first-order perceptual sensitivity (d’), second-order metacognitive sensitivity (meta-d’), and metacognitive efficiency (meta-d’/d’) for each participant in each sensory modality. To do this, we used a single-subject Bayesian estimation approach, which tends to perform better than the maximum likelihood estimation and sum-of-squared error approaches when there are relatively few trials per subject and condition (Fleming, 2017). We calculated metacognitive bias as the participant’s mean confidence rating in each task, irrespective of accuracy. Then we used Bayesian repeated measures ANOVAs and Bayesian paired samples *t*-tests to look for differences in perceptual sensitivity, metacognitive sensitivity, metacognitive efficiency, and mean confidence between sensory modalities.

We used Bayesian Pearson correlations with a default stretched beta prior over positive coefficient values (width = 1) to investigate whether individual differences in these four dependent variables were positively correlated across all possible pairs of sensory modalities in our design. For each condition and dependent measure, we report the mean and the 95% credible interval (CI). We used frequentist Steiger’s *Z* tests implemented by the R package cocor (Diedenhofen & Musch, 2015) to compare correlation coefficients for overlapping pairs of dependent measures. Additionally, we used a hierarchical Bayesian model to estimate group-level correlation coefficients for individual differences in metacognitive efficiency (Fleming, 2017).

All Bayesian hypothesis tests were performed in JASP (version 0.8.1.1; http://www.jasp-stats.org). BF_10_ values indicate how much more likely the alternative hypothesis is than the null hypothesis, given the prior and the evidence (Wagenmakers, Lodewyckx, Kuriyal, & Grasman, 2010). A BF_10_ greater than 3.00 or less than 0.33 is considered to show moderate support for the alternative or the null hypothesis, respectively. Similarly, a BF_10_ greater than 10.00 (or less than 0.10) is considered to show strong support for the alternative (or the null) hypothesis (Jeffreys, 1961, Lee and Wagenmakers, 2013). One of the main advantages of Bayesian hypothesis testing is that, unlike the *p*-value in standard frequentist hypothesis testing, the Bayes factor distinguishes between results that support the null hypothesis (BF_10_ < 0.33) and tests that lack the statistical power to infer support for either the alternative or the null hypothesis (0.33 < BF_10_ < 3.00). Thus, when reporting the results of these tests below, we distinguish between tests showing evidence for a difference (or correlation) between conditions (BF_10_ > 3.00), tests showing evidence for *no* difference (or correlation) between conditions (BF_10_ < 0.33), and tests that were inconclusive (0.33 < BF_10_ < 3.00).

## 3 Results

### 3.1 First-order performance

#### 3.1.1 Percentage of correct responses

A Bayesian repeated measures ANOVA showed strong evidence for differences in the percentage of correct responses between sensory modalities, BF_10_ = 1.04 × 10^7^. Follow-up Bayesian paired samples *t*-tests showed that participants made fewer correct responses in the innocuous warmth discrimination task (M = 68.9%, 95% CI = [67.6%, 70.1%]) than in the visual contrast discrimination task (M = 71.7%, 95% CI = [71.3%, 72.2%]), BF_10_ = 328, and the nociceptive pain discrimination task (M = 72.2%, 95% CI = [71.7%, 72.7%]), BF_10_ = 5.09 × 10^4^. The comparison between percentages of correct responses in the visual contrast discrimination task and the nociceptive pain discrimination task was inconclusive, BF_10_ = 0.47. These results indicate that our attempt to hold task difficulty constant across the three sensory modalities was not entirely successful. We placed a strict upper limit of 43.0 °C on the test stimulus in the innocuous warmth intensity staircase so that it would not increase into the noxious heat range. However, some participants gave incorrect answers even at the maximum temperature of the warm test stimulus, so overall performance in this modality was slightly worse than in the other two modalities. Such small but reliable differences in performance reinforce the need to appropriately control for perceptual sensitivity when quantifying metacognition.

#### 3.1.2 Perceptual sensitivity (d’)

**Fig. 2:**
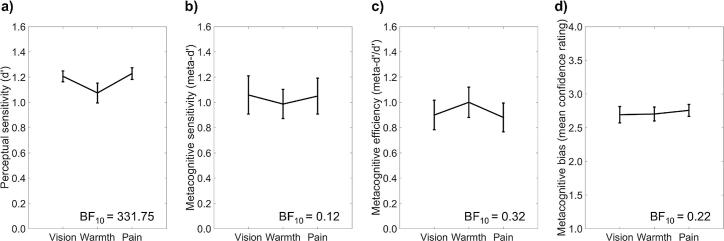
Mean values of (a) perceptual sensitivity, i.e. d’, (b) metacognitive sensitivity, i.e. meta-d’, (c) metacognitive efficiency, i.e. meta-d’/d’, and (d) metacognitive bias, i.e. mean confidence, in the visual contrast, innocuous warmth, and nociceptive pain discrimination tasks. A Bayes factor (BF_10_) > 3.00 indicates differences between conditions. A BF_10_ < 0.33 indicates *no* differences between conditions. Error bars show 95% credible intervals (CI).

A Bayesian repeated measures ANOVA also showed strong evidence for differences in perceptual sensitivity (d’) between sensory modalities, BF_10_ = 331.75. Follow-up Bayesian paired samples *t*-tests showed that perceptual sensitivity was lower in the innocuous warmth discrimination task (M = 1.08, 95% CI = [1.00, 1.15]) than in the visual contrast discrimination task (M = 1.21, 95% CI = [1.16, 1.25]), BF_10_ = 8.98, and the nociceptive pain discrimination task (M = 1.23, 95% CI = [1.18, 1.28]), BF_10_ = 74.90. There was no difference between perceptual sensitivity in the pain discrimination task and the visual discrimination task, BF_10_ = 0.24 (Fig. 2a). This pattern of results mirrors the differences in the percentage of correct responses between modalities (see above).

**Fig. 3:**
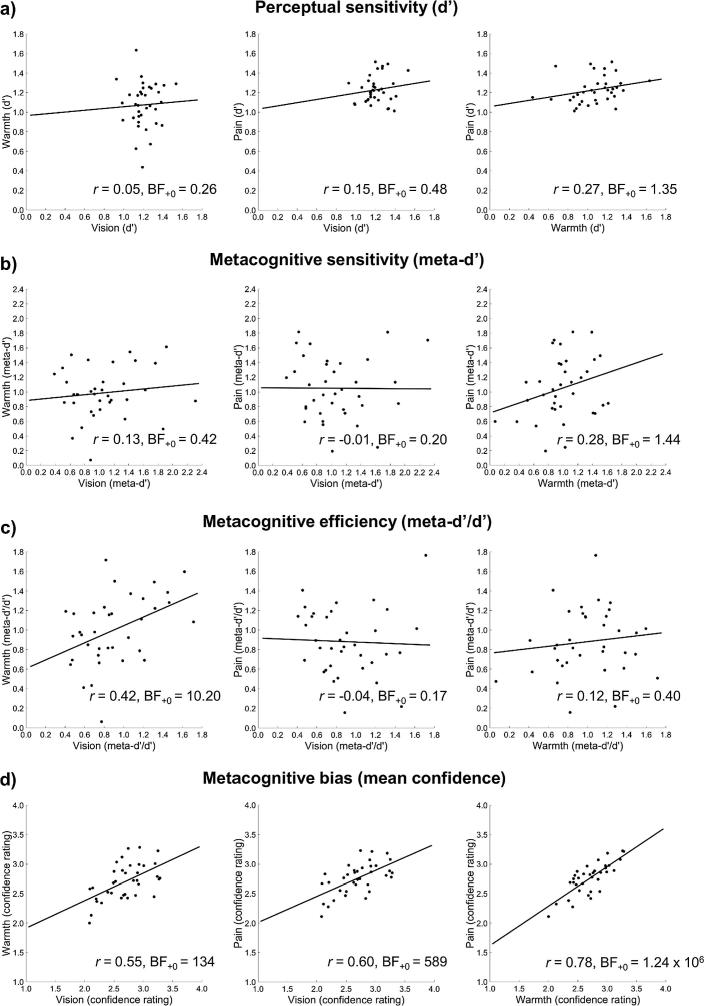
Correlations between modalities in (a) perceptual sensitivity, i.e. d’, (b) metacognitive sensitivity, i.e. meta-d’, (c) metacognitive efficiency, i.e. meta-d’/d’, and (d) metacognitive bias, i.e. mean confidence. In each row, all possible pairwise correlations between modalities are shown. A Bayes factor (BF_+0_) > 3.00 indicates a positive correlation. A BF_+0_ < 0.33 indicates *no* positive correlation.

Bayesian Pearson correlations showed that individual differences in perceptual sensitivity were not positively correlated between the visual discrimination task and the warmth discrimination task, *r* = 0.05, BF_+0_ = 0.26. The correlations between the pain and visual discrimination tasks, *r* = 0.15, BF_+0_ = 0.48, and the pain and warmth discrimination tasks, *r* = 0.27, BF_+0_ = 1.35, were inconclusive (Fig. 3a).

### 3.2 Second-order (metacognitive) performance

#### 3.2.1 Metacognitive sensitivity (meta-d’)

A Bayesian repeated measures ANOVA indicated that there were no differences in metacognitive sensitivity (meta-d’) between sensory modalities, BF_10_ = 0.12 (Fig. 2b). Mean metacognitive sensitivity scores were 1.06 (95% CI = [0.91, 1.21]) for visual contrast intensity judgements, 0.99 (95% CI = [0.87, 1.10]) for innocuous warmth intensity judgements, and 1.05 (95% CI = [0.91, 1.19]) for nociceptive pain intensity judgements.

Bayesian Pearson correlations showed that individual differences in metacognitive sensitivity were not positively correlated between the visual discrimination task and the pain discrimination task, *r* = −0.01, BF_+0_ = 0.20. The correlations between the visual and warmth discrimination tasks, *r* = 0.13, BF_+0_ = 0.42, and the pain and warmth discrimination tasks, *r* = 0.28, BF_+0_ = 1.44, were inconclusive (Fig. 3b).

#### 3.2.2 Metacognitive efficiency (meta-d’/d’)

We considered that our measure of metacognitive sensitivity—meta-d’—might be confounded by differences in perceptual sensitivity between conditions, because the innocuous warmth discrimination task was more difficult than the nociceptive pain and visual contrast discrimination tasks (Fig. 2a). In contrast, metacognitive efficiency scores are not confounded by small differences in perceptual sensitivity between conditions, because they represent the ratio of metacognitive sensitivity to perceptual sensitivity (i.e. meta-d’/d’). Thus, metacognitive efficiency provides a more appropriate measure than metacognitive sensitivity for how well confidence tracked performance in each modality.

A Bayesian repeated measures ANOVA indicated that there were no differences in metacognitive efficiency (meta-d’/d’) between sensory modalities, BF_10_ = 0.32 (Fig. 2c). As a group, participants were close to metacognitive optimality, with metacognitive efficiency scores near 1 (vision: M = 0.90, 95% CI = [0.78, 1.02]; warmth: M = 1.00, 95% CI = [0.88, 1.12]; pain: M = 0.88, 95% CI = [0.77, 1.00]). That is, the d’ that provided the best fit to confidence ratings was similar to observed perceptual sensitivity. This implies that there was no loss of (or gain in) perceptual information between the first-order perceptual decision and the second-order confidence judgement.

Bayesian Pearson correlations showed strong evidence that individual differences in metacognitive efficiency were positively correlated between visual discrimination and warmth discrimination tasks, *r* = 0.42, BF_+0_ = 10.20. (Note that we found evidence supporting the absence of a positive correlation between first-order visual and warmth discrimination performance, i.e. d’, so confounds with perceptual sensitivity cannot explain this finding.) Further correlation tests indicated no positive correlation between metacognitive efficiency scores in the visual discrimination task and the pain discrimination task, *r* = −0.04, BF_+0_ = 0.17. The correlation between the warmth and pain discrimination tasks was low, but inconclusive, *r* = 0.12, BF_+0_ = 0.40 (Fig. 3c).

Our Bayesian correlation tests showed strong evidence for a positive correlation between metacognitive efficiency scores in the visual and warmth discrimination tasks, and moderate evidence *against* a positive correlation between metacognitive efficiency scores in the visual and pain discrimination tasks. However, those tests did not directly compare the correlation coefficients to each other. To test for differences between correlation coefficients, we used two-tailed Steiger’s *Z* tests for overlapping correlations (employing a standard frequentist hypothesis-testing approach). We found a significant difference between the vision-warmth and vision-pain correlations, *Z* = 2.13, *p* = 0.033. This further supports the finding of greater shared variance in metacognitive efficiency between the visual and warmth discrimination tasks than between the visual and pain discrimination tasks. Comparisons between vision-warmth and pain-warmth correlations, *Z* = 1.29, *p* = 0.198, and between vision-pain and pain-warmth correlations, *Z* = −0.89, *p* = 0.372, were not significant. (Note that frequentist hypothesis tests do not distinguish between evidence for the absence of a difference and insufficient statistical power to detect a difference.)

All preceding correlation tests were based on point estimates of metacognitive efficiency from a relatively small number of participants (*N* = 36). Single-subject estimates of metacognitive efficiency can be noisy, so our estimates of the correlation coefficients may have also been imprecise. To overcome this potential issue, we used a hierarchical Bayesian model to estimate the covariance in metacognitive efficiency between visual, warmth, and pain discrimination tasks. A hierarchical Bayesian model ensures that uncertainty in subject-level parameter estimates appropriately propagates through to uncertainty around estimates of cross-task covariance (Fleming, 2017). In this case, the hierarchical model fits revealed the same pattern of results as the single-subject estimates. There was a significant positive correlation in individual differences in metacognitive efficiency between the visual and warmth discrimination tasks, ρ = 0.69, 95% CI = [0.06, 0.98]. (Note that statistical significance is obtained when the 95% CI does not overlap with zero.) Individual differences in metacognitive efficiency were not correlated between the visual and pain discrimination tasks, ρ = −0.02, 95% CI = [−0.71, 0.87]. The coefficient for the correlation between the warmth and pain discrimination tasks was moderately positive but inconclusive, as the 95% CI overlapped with zero, ρ = 0.35, 95% CI = [−0.48, 0.97].

In all three tasks, several participants had metacognitive efficiency values greater than 1 (Fig. 3c), indicating higher metacognitive sensitivity (meta-d’) than perceptual sensitivity (d’). This might occur if confidence depended on some processes independent of performance, for example processes that occur after decision, or in parallel to decision-making (Fleming & Daw, 2017). However, both d’ and meta-d’ estimates are inevitably subject to error. Metacognitive efficiency, as the ratio of the latter to the former, will be influenced by these errors, particularly when d’ is low. We therefore also examined an alternative measure of metacognitive efficiency, meta-d’−d’, which is less prone to such error amplification. This alternative measure yielded similar results (see Supplementary Results and Fig. S1).

#### 3.2.3 Metacognitive bias (mean confidence)

A Bayesian repeated measures ANOVA indicated that there were no differences in metacognitive bias (mean confidence) between sensory modalities, BF_10_ = 0.22 (Fig. 2d). Mean confidence ratings were 2.69 (95% CI = [2.57, 2.81]) for visual contrast intensity judgements, 2.70 (95% CI = [2.60, 2.81]) for innocuous warmth intensity judgements, and 2.76 (95% CI = [2.67, 2.84]) for nociceptive pain intensity judgements.

Bayesian Pearson correlations showed strong evidence that individual differences in metacognitive bias were positively correlated across all three sensory modalities (vision and warmth: *r* = 0.55, BF_+0_ = 134; vision and pain: *r* = 0.60, BF_+0_ = 589; warmth and pain: *r* = 0.78, BF_+0_ = 1.24 × 10^6^; Fig. 3d).

### 3.3 Manipulation check for thermal stimuli

**Fig. 4:**
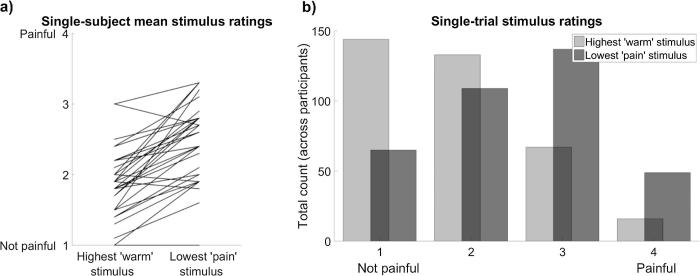
Variability in participants’ ratings of the highest level of stimulation used in the innocuous warmth discrimination task (max. 43.0 °C) and the lowest level of stimulation used in the nociceptive pain discrimination task (always 45.0 °C). Overall, the lowest level of noxious heat was perceived as more painful than the highest level of innocuous warmth. However, perception of these stimuli varied both (a) between participants and (b) between trials.

A Bayesian paired samples *t*-test showed strong evidence that participants felt a difference between the lowest level of noxious heat stimulation and the highest level of innocuous warmth stimulation delivered on any trial, BF_10_ = 1.24 × 10^7^, thus validating that the lowest temperature stimulus in the noxious heat range was rated as more painful (M = 2.47, 95% CI = [2.29, 2.65]) than the highest temperature stimulus in the innocuous warmth range (M = 1.88, 95% CI = [1.71, 2.04]). There was, however, some variability in how the stimuli were perceived, both between and within individuals (Fig. 4). This was expected, yet we were not able to further separate the temperature ranges we used for the innocuous warmth and nociceptive pain discrimination tasks, due to the maximum safe contact heat temperature of 50.0 °C, and the need to control first-order performance by varying the temperature difference between stimuli in a staircase procedure. We consider the implications of this design limitation in the Discussion. Importantly, our results do not change if we exclude the four participants who did not rate the lowest level of noxious heat as more painful than the highest level of innocuous warmth (see Fig. 4a and Supplementary Results).

## 4 Discussion

Our results do not support the hypothesis of reduced metacognitive access to nociceptive pain and innocuous thermal perception, compared to vision. We found no overall differences in metacognitive efficiency (meta-d’/d’) between intensity judgements of visual contrast, innocuous warmth, and nociceptive pain (Fig. 2c). Some authors have proposed that interoceptive modalities lack the metacognitive sensitivity that accompanies exteroception (Azevedo et al., 2016, Garfinkel et al., 2015, Khalsa et al., 2008). Like interoceptive senses, the primary functions of both thermoceptive and nociceptive sensory systems are to maintain the optimal condition of the body and to defend it from harm (Craig, 2002, Craig, 2003). The visual system, on the other hand, allows us to make fine discriminative judgements about objects and events in our surroundings. The processes of cognitive control and flexible behaviour enabled by metacognition (Redford, 2010, Yeung and Summerfield, 2012) might better serve discriminative functions than regulatory or defensive functions, the latter of which must operate effectively without conscious oversight. Nevertheless, our study indicates comparable metacognitive access to both discriminative and regulatory sensory modalities.

Moreover, we found that individual differences in metacognitive efficiency were positively correlated between the visual contrast and innocuous warmth discrimination tasks (Fig. 3c). Importantly, that correlation must have arisen from individual differences in metacognition rather than first-order perception, because there was no correlation in first-order perceptual sensitivity (d’) between the same tasks (Fig. 3a). This finding suggests there is a common metacognitive system for vision and innocuous thermal perception, despite their disparate roles in fine discrimination of stimulus attributes and regulation of the body’s condition, respectively. A previous study found no correlation in metacognitive sensitivity between a discriminative sense (touch) and regulatory, interoceptive senses (cardiac and respiratory signals), suggesting distinct metacognitive processes for those sensory categories (Garfinkel et al., 2016). However, those authors used a measure of metacognitive sensitivity—the type II ROC curve—that is potentially confounded by perceptual task performance. Our measure of metacognitive efficiency is not subject to such confounds (Fleming & Lau, 2014).

Conversely, we found evidence *against* the existence of a correlation between metacognitive efficiency for vision and nociception (Fig. 3c). Further, we found little evidence of a correlation in metacognitive efficiency between nociception and innocuous thermoception, even though the two are similar in terms of their functional roles and physiological pathways (Craig, 2002, Craig, 2003). This is particularly striking because we used the same equipment and procedure to administer the stimuli for the innocuous warmth and nociceptive pain discrimination tasks, except that the thermal probe temperature was increased into the noxious heat range in the latter task. The unshared variance in nociceptive metacognition was not predicted, and awaits further support from replication studies. Nevertheless, we consider that it could either reflect a distinct metacognitive process, or an additional source of variation due to individual differences in some component that accompanies pain, such as affect or arousal responses. Pain has a strong affective component in addition to its sensory component (Melzack & Casey, 1968). Ratings of pain intensity and unpleasantness can even be dissociated, (e.g. Gracely et al., 1979, Rainville et al., 1999, Smith et al., 1998), suggesting that affect is a distinctive component of pain, rather than a mere by-product. In our nociceptive pain discrimination task, participants reported which of two noxious heat stimuli was more painful without being asked to focus on either sensory or affective aspects, so their judgements presumably reflected both these components of pain. Moreover, pain can produce physiological arousal responses (Hilgard and Morgan, 1975, Lenox, 1970, Rainville et al., 1999, Storm, 2008), another factor known to influence metacognition (Allen et al., 2016, Hauser et al., 2017). Since noxious heat stimuli are both more arousing and more negatively valenced than innocuous thermal or visual contrast stimuli, these potential sources of variability would have been stronger in the nociceptive pain discrimination task than in the other tasks. Either the affective or arousal components of pain may thus have contributed to the unshared variance in nociceptive metacognition that we found here.

In all three discrimination tasks, there were several participants with metacognitive efficiency (meta-d’/d’) values greater than 1 (Fig. 3c). Such a finding could potentially result from imprecise estimates of low values of d’. Although there were a few outliers with low d’ values in the warmth discrimination task (Fig. 3a), for the most part, our staircase procedure yielded sufficiently high levels of d’ to avoid this problem. Moreover, we analysed our data using an alternative, non-ratio measure of metacognitive efficiency (meta-d’−d’), and found the same results (see Supplementary Results and Fig. S1). Thus, our finding suggests that some participants experienced a *gain* in confidence-related information between their first-order perceptual decision and their subsequent, second-order confidence rating. Some previous studies that measured metacognitive efficiency have also found this trend (Charles et al., 2013, Faivre et al., 2018). One possible explanation is that parallel accumulation of evidence or post-decisional processing allowed the recognition of errors in first-order decisions (Charles et al., 2013, Fleming and Daw, 2017). Our use of unspeeded perceptual judgements should have mitigated this influence by reducing errors related to quick responses. Nonetheless, given the difficulty of the discriminations they were asked to make, some participants may have changed their minds after their first decision and assigned lower confidence ratings to trials where they made an error, resulting in higher metacognitive sensitivity (meta-d’) than perceptual sensitivity (d’).

In addition, we examined metacognitive bias across vision, innocuous warmth, and nociceptive pain perception. There were no overall differences in confidence between modalities (Fig. 2d), and individual differences in mean confidence ratings were highly correlated across all three tasks (Fig. 3d). This is consistent with previous studies that found correlations in mean confidence levels across different tasks, both within and between sensory modalities (Ais et al., 2016, Song et al., 2011) and between perceptual and memory domains (Baird et al., 2015, Baird et al., 2013, McCurdy et al., 2013). Some studies also found a task-dependent component of metacognitive bias which was attributed to differences in difficulty between tasks (Baird et al., 2015, Baird et al., 2013, Song et al., 2011). We did not find a task-dependent component of metacognitive bias, even though the innocuous warmth discrimination task was more difficult than the nociceptive pain discrimination task and the visual contrast discrimination task. Thus, our participants did not adjust their average confidence reports according to task difficulty. In this study, at least, consistent individual differences in confidence were the strongest contributing factor to metacognitive bias.

Altogether, the results of our correlation tests suggest that metacognition consists of both a modality-independent component (i.e. metacognitive bias) and a modality-dependent component (i.e. metacognitive efficiency). The former was a consistent trait of individuals, while the latter differentiated judgements about nociceptive pain. Further, our findings suggest that metacognitive ability does not dissociate between senses serving primarily regulatory or discriminative functions, as has been previously suggested for interoceptive and exteroceptive somatosensory modalities (Garfinkel et al., 2016). However, our results also refute pure modality-specificity in metacognitive ability, whereby individual differences in metacognitive efficiency would not correlate across any sensory modalities.

**Fig. 5:**
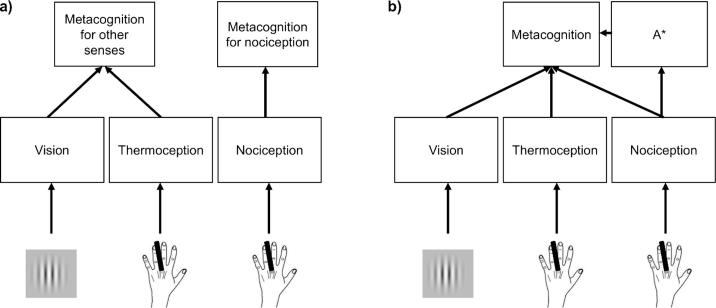
The distinctive variance in nociceptive metacognition within our design could come from either (a) a separate metacognitive process for nociception, or (b) an additional processing operation (A^*^), uniquely or disproportionately engaged by noxious stimulation, that also contributes to a supramodal metacognitive process.

Confidence is often modelled as the strength or quality of the evidence that contributes to a first-order decision (Kepecs et al., 2008, Kiani and Shadlen, 2009, Merkle and Van Zandt, 2006). However, it is unclear how first-order models could account for differences in covariance of metacognitive ability across modalities, as we observed here. In contrast, hierarchical models conceptualise metacognition as a distinct second-order network that represents and evaluates the state of the first-order network computing the decision (Cleeremans et al., 2007, Fleming and Daw, 2017, Pasquali et al., 2010). Such models might explain our results in two ways. Under one account, metacognitive ability might be correlated when sensory evidence for two different modalities converges on a single metacognitive monitoring process. This account might predict a distinct metacognitive monitoring process for nociception—although why this separate circuit should have evolved remains unclear (Fig. 5a). Alternatively, as we mentioned above, there might be a single metacognitive mechanism for all sensory modalities, but this mechanism might be differentially affected by non-sensory inputs such as arousal or affect. Modalities that differ sharply in their recruitment of these additional factors would also exhibit low correlations in metacognitive ability (Fig. 5b).

Definitions of pain routinely insist on its subjective nature, and some hold the view that pain can never have any ‘ground truth’ in the physical properties of the world. Chronic pain conditions, which sometimes lack any apparent neurophysiological aetiology, might encourage this view. In our study, however, participants made judgements about pain that directly resulted from noxious thermal stimulation of nociceptive sensory pathways. Moreover, the 2IFC intensity discrimination task we used was specifically designed to test a discriminative aspect of nociceptive pain, similarly to our tests of innocuous warmth and visual contrast discrimination. By applying signal detection theory, we could determine how much participants’ pain reports were informed by the properties of the evoking stimulus (i.e. the first-order judgement), as well as how people experienced the processes that contributed to the formation of their pain reports (i.e. the second-order judgement, captured here using the established method of confidence ratings). This method allowed us to investigate the relation between judgements about experimentally evoked pain and underlying nociceptive processes, without insisting that pain is reducible to nociception. An alternative approach could have been to ask participants to report which noxious stimulus was hotter, rather than which was more painful. Such an instruction may have induced them to focus on the thermal quality of the noxious stimulation instead of its painfulness. The potential impact of this manipulation on our findings is an open question, and would depend upon whether the unshared variance in metacognitive efficiency for nociceptive pain came from the noxious nature of the stimulus, or from the task requirement to judge pain levels.

One limitation of our study was an inability to adjust the temperature ranges of innocuous warmth and noxious heat stimulation so that, for every participant, the latter always felt painful and the former never felt painful at all. We were constrained by safety considerations, which placed an upper limit of 50.0 °C on contact thermal stimulation. Additionally, we were constrained by the need to adapt the intensity of the test stimulus throughout the task, so that we could control first-order task performance and specifically test differences between modalities at the metacognitive level. For the innocuous warmth discrimination task, in particular, this often required a large difference between stimulus temperatures. Thus, we could not further separate the innocuous and noxious temperature ranges without compromising these important considerations, even though it meant that participants would sometimes perceive the upper end of the innocuous warmth range as somewhat painful, or the lower end of the noxious heat range as not at all painful (Fig. 4). If, as we speculate above, the unshared variance in metacognitive efficiency for nociceptive pain judgements arose from affective or arousal responses to noxious stimulation, then we might have found a clearer dissociation between metacognitive efficiency for innocuous warmth and nociceptive pain discrimination if we had adjusted the temperature ranges used for each individual participant based on their painfulness. It is also possible that confidence in judgements about nociceptive pain intensity could be substantively different when discriminating a painful stimulus and a non-painful stimulus, compared to two painful stimuli. We cannot exclude the possibility that some trials in our nociceptive pain discrimination task involved comparing stimuli of different *quality* (painful vs non-painful) rather than comparing stimuli of different *intensity* (more vs less painful). This may have introduced some variance in metacognitive efficiency that was not shared with the other tasks. Future studies could explore these issues by using innocuous and noxious thermal stimulation parameters that separate more clearly along the dimension of painfulness (e.g. innocuous cool temperatures vs noxious heat stimuli).

To conclude, we demonstrated that confidence tracks perceptual intensity judgements as precisely for nociceptive pain as for other modalities. However, we found no correlation between metacognitive efficiency for nociception and for vision, and minimal correlation between metacognitive efficiency for nociception and for thermoception. Thus, second-order judgements about nociceptive pain level appear to involve an additional factor, which may be the arousal and/or affective responses typical of noxious stimulation. Metacognitive appraisal is closely linked to higher-order accounts of conscious experience (Lau & Rosenthal, 2011). Our findings are thus consistent with the interesting possibility that distinctive and idiosyncratic features of the nociceptive pain experience, namely high vividness and inter-individual variability, may lie in the affective or motivational components of pain rather than the sensory component.

## Footnotes

2 Note that none of those findings were based exclusively on the heartbeat counting task, which was shown to be a flawed measure of interoceptive accuracy (Zamariola, Maurage, Luminet, & Corneille, 2018).

## References

[b0005] Ais J., Zylberberg A., Barttfeld P., Sigman M. (2016). Individual consistency in the accuracy and distribution of confidence judgments. Cognition.

[b0010] Allen M., Frank D., Schwarzkopf D.S., Fardo F., Winston J.S., Hauser T.U., Rees G. (2016). Unexpected arousal modulates the influence of sensory noise on confidence. Elife.

[b0015] Azevedo R.T., Aglioti S.M., Lenggenhager B. (2016). Participants' above-chance recognition of own-heart sound combined with poor metacognitive awareness suggests implicit knowledge of own heart cardiodynamics. Scientific Reports.

[b0020] Baird B., Cieslak M., Smallwood J., Grafton S.T., Schooler J.W. (2015). Regional white matter variation associated with domain-specific metacognitive accuracy. Journal of Cognitive Neuroscience.

[b0025] Baird B., Smallwood J., Gorgolewski K.J., Margulies D.S. (2013). Medial and lateral networks in anterior prefrontal cortex support metacognitive ability for memory and perception. Journal of Neuroscience.

[b0030] Beecher H.K. (1957). The measurement of pain: Prototype for the quantitative study of subjective responses. Pharmacological Reviews.

[b0035] Beecher H.K. (1965). Quantification of the subjective pain experience. Proceeding of the Annual Meeting of the American Psychopathological Association.

[b0040] Charles L., Van Opstal F., Marti S., Dehaene S. (2013). Distinct brain mechanisms for conscious versus subliminal error detection. NeuroImage.

[b0045] Cleeremans A., Timmermans B., Pasquali A. (2007). Consciousness and metarepresentation: A computational sketch. Neural Networks.

[b0050] Coghill R.C., McHaffie J.G., Yen Y.F. (2003). Neural correlates of interindividual differences in the subjective experience of pain. Proceeding of the National Academy of Sciences of the United States of America.

[b0055] Craig A.D. (2002). How do you feel? Interoception: The sense of the physiological condition of the body. Nature Reviews Neuroscience.

[b0060] Craig A.D. (2003). Interoception: The sense of the physiological condition of the body. Current Opinion in Neurobiology.

[b0065] D'Angelo M.C., Humphreys K.R. (2012). Emotional cues do not increase the likelihood of tip-of-the-tongue states. Memory & Cognition.

[b0070] Diedenhofen B., Musch J. (2015). cocor: A comprehensive solution for the statistical comparison of correlations. PLoS One.

[b0075] Dyck P.J., Zimmerman I., Gillen D.A., Johnson D., Karnes J.L., O'Brien P.C. (1993). Cool, warm, and heat-pain detection thresholds: Testing methods and inferences about anatomic distribution of receptors. Neurology.

[b0080] Ellrich J., Bromm B., Hopf H.C. (1997). Pain-evoked blink reflex. Muscle and Nerve.

[b0085] Faivre N., Filevich E., Solovey G., Kühn S., Blanke O. (2018). Behavioral, modeling, and electrophysiological evidence for supramodality in human metacognition. Journal of Neuroscience.

[b0090] Fleming S.M. (2017). HMeta-d: Hierarchical Bayesian estimation of metacognitive efficiency from confidence ratings. Neuroscience of Consciousness.

[b0095] Fleming S.M., Daw N.D. (2017). Self-evaluation of decision-making: A general Bayesian framework for metacognitive computation. Psychological Review.

[b0100] Fleming S.M., Lau H.C. (2014). How to measure metacognition. Frontiers in Human Neuroscience.

[b0105] Garfinkel S.N., Manassei M.F., Hamilton-Fletcher G., In den Bosch Y., Critchley H.D., Engels M. (2016). Interoceptive dimensions across cardiac and respiratory axes. Philosophical Transactions of the Royal Society London B Biological Science.

[b0110] Garfinkel S.N., Seth A.K., Barrett A.B., Suzuki K., Critchley H.D. (2015). Knowing your own heart: Distinguishing interoceptive accuracy from interoceptive awareness. Biological Psychology.

[b0115] Gracely R.H., Dubner R., McGrath P.A. (1979). Narcotic analgesia: Fentanyl reduces the intensity but not the unpleasantness of painful tooth pulp sensations. Science.

[b0120] Guerit J.M. (2012). Neurophysiological pain assessment: How to objectify a subjective phenomenon?. Neurophysiology Clinical.

[b0125] Hauser T.U., Allen M., Purg N., Moutoussis M., Rees G., Dolan R.J. (2017). Noradrenaline blockade specifically enhances metacognitive performance. Elife.

[b0130] Hilgard E.R., Morgan A.H. (1975). Heart rate and blood pressure in the study of laboratory pain in man under normal conditions and as influenced by hypnosis. Acta Neurobiologiae Experimentalis (Wars).

[b0135] Hyyppä M.T. (1987). The understanding and evaluation of chronic pain: Subjective vs. objective determinants of pain. International Journal of Rehabilitation Research.

[b0140] IASP Task Force on Taxonomy, Merskey H., Bogduk N. (2011). Part III: Pain terms, a current list with definitions and notes on usage. Classification of chronic pain (second edition, revised).

[b0145] Jeffreys H.S. (1961). Theory of probability.

[b0150] Jersakova R., Souchay C., Allen R.J. (2015). Negative affect does not impact semantic retrieval failure monitoring. Canadian Journal of Experimental Psychology-Revue Canadienne De Psychologie Experimentale.

[b0155] Kepecs A., Uchida N., Zariwala H.A., Mainen Z.F. (2008). Neural correlates, computation and behavioural impact of decision confidence. Nature.

[b0160] Khalsa S.S., Rudrauf D., Damasio A.R., Davidson R.J., Lutz A., Tranel D. (2008). Interoceptive awareness in experienced meditators. Psychophysiology.

[b0165] Kiani R., Shadlen M.N. (2009). Representation of confidence associated with a decision by neurons in the parietal cortex. Science.

[b0170] Koizumi A., Mobbs D., Lau H. (2016). Is fear perception special? Evidence at the level of decision-making and subjective confidence. Social Cognitive and Affect Neuroscience.

[b0175] Koyama T., McHaffie J.G., Laurienti P.J., Coghill R.C. (2005). The subjective experience of pain: Where expectations become reality. Proceeding of the National Academy of Sciences of the United States of America.

[b0180] Lau H., Rosenthal D. (2011). Empirical support for higher-order theories of conscious awareness. Trends in Cognitive Sciences.

[b0185] Lee M.D., Wagenmakers E.-J. (2013). Bayesian cognitive modeling: A practical course.

[b0190] Lenox J.R. (1970). Effect of hypnotic analgesia on verbal report and cardiovascular responses to ischemic pain. Journal of Abnormal Psychology.

[b0195] Levitt H. (1971). Transformed up-down methods in psychoacoustics. Journal of the Acoustical Society of America.

[b0200] Maniscalco B., Lau H. (2012). A signal detection theoretic approach for estimating metacognitive sensitivity from confidence ratings. Consciousness and Cognition.

[b0205] McCurdy L.Y., Maniscalco B., Metcalfe J., Liu K.Y., de Lange F.P., Lau H. (2013). Anatomical coupling between distinct metacognitive systems for memory and visual perception. Journal of Neuroscience.

[b0210] Melzack R., Casey K.L., Kenshalo D. (1968). Sensory, motivational, and central control determinants of pain: A new conceptual model. International symposium on the skin senses.

[b0215] Merkle E.C., Van Zandt T. (2006). An application of the poisson race model to confidence calibration. Journal of Experimental Psychology: General.

[b0220] Metcalfe J., Shimamura A.P. (1994). Metacognition: Knowing about knowing.

[b0225] Nickel M.M., May E.S., Tiemann L., Schmidt P., Postorino M., Dinh S.T., Ploner M. (2017). Brain oscillations differentially encode noxious stimulus intensity and pain intensity. NeuroImage.

[b0230] Pasquali A., Timmermans B., Cleeremans A. (2010). Know thyself: Metacognitive networks and measures of consciousness. Cognition.

[b0235] Raij T.T., Numminen J., Narvanen S., Hiltunen J., Hari R. (2005). Brain correlates of subjective reality of physically and psychologically induced pain. Proceeding of the National Academy of Sciences of the United States of America.

[b0240] Rainville P., Carrier B., Hofbauer R.K., Bushnell M.C., Duncan G.H. (1999). Dissociation of sensory and affective dimensions of pain using hypnotic modulation. Pain.

[b0245] Redford J.S. (2010). Evidence of metacognitive control by humans and monkeys in a perceptual categorization task. Journal of Experimental Psychology: Learning, Memory, and Cognition.

[b0250] Schönbrodt F.D., Wagenmakers E.J., Zehetleitner M., Perugini M. (2017). Sequential hypothesis testing with Bayes factors: Efficiently testing mean differences. Psychological Methods.

[b0255] Schulz E., May E.S., Postorino M., Tiemann L., Nickel M.M., Witkovsky V., Ploner M. (2015). Prefrontal gamma oscillations encode tonic pain in humans. Cerebral Cortex.

[b0260] Schwartz B.L. (2010). The effects of emotion on tip-of-the-tongue states. Psychonomic Bulletin Review.

[b0265] Skljarevski V., Ramadan N.M. (2002). The nociceptive flexion reflex in humans—reveiew article. Pain.

[b0270] Smith W.B., Gracely R.H., Safer M.A. (1998). The meaning of pain: Cancer patients' rating and recall of pain intensity and affect. Pain.

[b0275] Song C., Kanai R., Fleming S.M., Weil R.S., Schwarzkopf D.S., Rees G. (2011). Relating inter-individual differences in metacognitive performance on different perceptual tasks. Consciousness and Cognition.

[b0280] Storm H. (2008). Changes in skin conductance as a tool to monitor nociceptive stimulation and pain. Current Opinion in Anaesthesiology.

[b0285] Wagenmakers E.J., Lodewyckx T., Kuriyal H., Grasman R. (2010). Bayesian hypothesis testing for psychologists: A tutorial on the Savage-Dickey method. Cognitive Psychology.

[b0290] Willer J.C. (1977). Comparative study of perceived pain and nociceptive flexion reflex in man. Pain.

[b0295] Woo C.-W., Schmidt L., Krishnan A., Jepma M., Roy M., Lindquist M.A., Wager T.D. (2017). Quantifying cerebral contributions to pain beyond nociception. Nature Communications.

[b0300] Yarnitsky D., Sprecher E., Zaslansky R., Hemli J.A. (1995). Heat pain thresholds: Normative data and repeatability. Pain.

[b0305] Yeung N., Summerfield C. (2012). Metacognition in human decision-making: Confidence and error monitoring. Philosophical Transactions of the Royal Society London B Biological Science.

[b0310] Zamariola G., Maurage P., Luminet O., Corneille O. (2018). Interoceptive accuracy scores from the heartbeat counting task are problematic: Evidence from simple bivariate correlations. Biological Psychology.

[b0315] Zimmerman C.A., Kelley C.M. (2010). “I'll remember this!” Effects of emotionality on memory predictions versus memory performance. Journal of Memory and Language.

