# Supplementary material for: Metacognition across sensory modalities: Vision, warmth, and nociceptive pain
Source: Cognition. 2019 May;186:32–41. doi: 10.1016/j.cognition.2019.01.018 (PMC6411924; doi:10.1016/j.cognition.2019.01.018)
Supplement: Supplementary data 1 [file mmc1.docx]

**1. Supplementary Results**

**1.1. Alternative measure of metacognitive efficiency (meta-d’−d’)**

A Bayesian repeated measures ANOVA indicated that there were no differences in our alternative measure of metacognitive efficiency (meta-d’−d’) between sensory modalities, BF_10_ = 0.15 (Fig. S1a). Bayesian Pearson correlations showed moderate evidence that individual differences in metacognitive efficiency were positively correlated between visual contrast discrimination and innocuous warmth discrimination tasks, *r* = 0.40, BF_+0_ = 6.69 (Fig. S1b). There was no positive correlation between metacognitive efficiency scores in the visual contrast discrimination task and the nociceptive pain discrimination task, *r* = -0.04, BF_+0_ = 0.18 (Fig. S1c). The correlation between the warmth and pain discrimination tasks was low, but inconclusive, *r* = 0.15, BF_+0_ = 0.47 (Fig. S1d). These results corroborate the results we obtained with our primary measure of metacognitive efficiency (meta-d’/d’), while avoiding the potential problem of using a ratio measure when perceptual sensitivity (d’) is low.


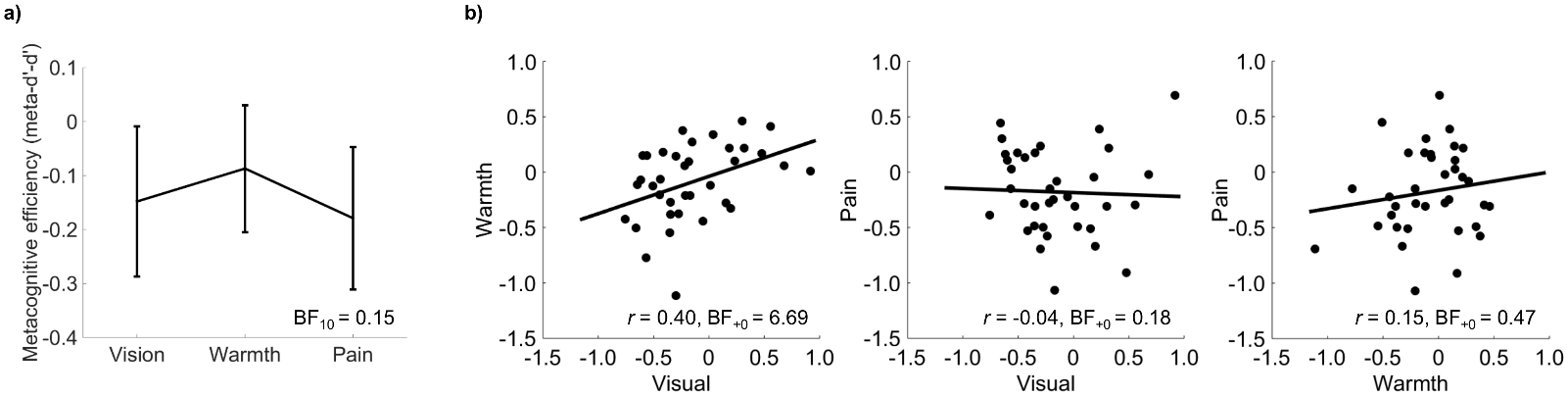


Figure *S1*. Results of analyses using an alternative, non-ratio measure of metacognitive efficiency: meta-d’−d’, including (a) mean metacognitive efficiency values in each modality, and (b) correlations in metacognitive efficiency between modalities. Note that the results corroborate those found using our primary measure of metacognitive efficiency, meta-d’/d’. Error bars show 95% credible intervals (CI).

To test for differences between correlation coefficients, we used two-tailed Steiger’s *Z* tests for overlapping correlations (employing a standard frequentist hypothesis testing approach). There was a significant difference between the vision-warmth and vision-pain correlations, *Z* = 2.01, *p* = .044. Comparisons between vision-warmth and pain-warmth correlations, *Z* = 1.07, *p* = .282, and between vision-pain and pain-warmth correlations, *Z* = -0.98, *p* = .327, were not significant. Again, these results corroborate those we obtained using a ratio measure of metacognitive efficiency.

**1.2 Metacognitive performance after excluding participants based on pain ratings**

Our manipulation check for thermal stimulation found four participants who did not rate the lowest level of noxious heat stimulation as more painful than the highest level of innocuous warmth stimulation. To determine whether any effects particular to those four participants could have driven our results, we excluded them and re-analysed metacognitive efficiency scores (meta-d’/d’). The analysis on this subset of 32 participants yielded the same results as the analysis of the full dataset. There was no difference in metacognitive efficiency between modalities, BF_10_ = 0.16. Moreover, individual differences in metacognitive efficiency were positively correlated between the visual contrast and innocuous warmth discrimination tasks, *r* = 0.38, BF_+0_ = 3.79, but not between the visual contrast and nociceptive pain discrimination tasks, *r* = -0.02, BF_+0_ = 0.20. The correlation between the warmth and pain discrimination tasks was still inconclusive, *r* = 0.16, BF_+0_ = 0.50.

We also re-analysed metacognitive bias (mean confidence) after excluding the four participants. Again, our results did not change. There was no difference in mean confidence ratings between modalities, BF_10_ = 0.21. Additionally, individual differences in mean confidence were positively correlated across all three sensory modalities (vision and warmth: *r* = 0.49, BF_+0_ = 22.35; vision and pain: *r* = 0.58, BF_+0_ = 150.36; warmth and pain: *r* = 0.78, BF_+0_ = 3.09 x 10^5^).
